# Unique Features of Ethnic Mongolian Gut Microbiome revealed by metagenomic analysis

**DOI:** 10.1038/srep34826

**Published:** 2016-10-06

**Authors:** Wenjun Liu, Jiachao Zhang, Chunyan Wu, Shunfeng Cai, Weiqiang Huang, Jing Chen, Xiaoxia XI, Zebin Liang, Qiangchuan Hou, Bing Zhou, Nan Qin, Heping Zhang

**Affiliations:** 1 Key Laboratory of Dairy Biotechnology and Engineering, Education Ministry of P. R. China, Department of Food Science and Engineering, Inner Mongolia Agricultural University, Hohhot 010018, China; 2 RealBio Genomic Institute, Shanghai 200050, China; 3 State Key Laboratory for Diagnosis and Treatment of Infectious Disease, Collaborative Innovation Center for Diagnosis and Treatment of Infectious Diseases, the First Affiliated Hospital, Zhejiang University, Hangzhou 310003, China

## Abstract

The human gut microbiota varies considerably among world populations due to a variety of factors including genetic background, diet, cultural habits and socioeconomic status. Here we characterized 110 healthy Mongolian adults gut microbiota by shotgun metagenomic sequencing and compared the intestinal microbiome among Mongolians, the Hans and European cohorts. The results showed that the taxonomic profile of intestinal microbiome among cohorts revealed the Actinobaceria and *Bifidobacterium* were the key microbes contributing to the differences among Mongolians, the Hans and Europeans at the phylum level and genus level, respectively. Metagenomic species analysis indicated that *Faecalibacterium prausnitzii* and *Coprococcus comes*were enrich in Mongolian people which might contribute to gut health through anti-inflammatory properties and butyrate production, respectively. On the other hand, the enriched genus *Collinsella*, biomarker in symptomatic atherosclerosis patients, might be associated with the high morbidity of cardiovascular and cerebrovascular diseases in Mongolian adults. At the functional level, a unique microbial metabolic pathway profile was present in Mongolian’s gut which mainly distributed in amino acid metabolism, carbohydrate metabolism, energy metabolism, lipid metabolism, glycan biosynthesis and metabolism. We can attribute the specific signatures of Mongolian gut microbiome to their unique genotype, dietary habits and living environment.

The gut microbiota (GM) is recognized as a human co-evolutionary partner that facilitates nutritional acquisition and immune modulation, and helps maintain host homeostasis in response to profound lifestyle changes^1^,^2^,^3^. Researchers have delineated the structural and functional configurations of gut metagenomes in different world populations by next generation sequencing^4^,^5^,^6^,^7^. The functional GM layout may represent host genotypic characteristics and reflect an adaptive ecosystem response to the host diet and cultural habits.

The microbiota of the Mongolian population is of particular interest to researchers, because Mongolia encompasses a uniquely wide range of environmental conditions and ethnogeographical cohorts. Moreover, the Mongol Empire was the world’s largest contiguous empire, exerted a major influence that greatly enhanced the cultural exchange between Mongolia and Europe that took place during the Middle Ages, when Mongolians intermingled with various populations in the Eurasian continent. Importantly, it has been reported that genetically, more than 20% people in the world are related to Mongolians^8^. Mongolians living in pastureland, such as Khentii Province, maintains a traditional nomadic lifestyle (Fig. 1A), whereas those in urban areas, such as Ulan Bator (the capital of Mongolia) and TUW Province (the suburbs of the capital), have adopted an urban lifestyle due to rapid modernization and economic development. Chinese Mongolians living in Hohhot and Xilingol have inter-married with ethic Han Chinese, which may generate some differences from their brethren in the north at the genomic level. Nevertheless, all ethnic Mongolians consume similar diet, including traditional cheese (Fig. 1B) and red meat (Fig. 1C) as their main food.

In our previous study, by employing 454 pyrosequencing technology, we described the profiles of gut microbiota of Mongolians from Mongolia and China. At the genus level, *Prevotella* of the *Firmicutes* phylum was the most abundant genus, and the amounts of *Bacteroides, Faecalibacterium, Oscillibacter, Roseburia, Clostridium, Coprococcus, Ruminococcus, Alistipes, Parabacteroides, Catenibacterium, Subdoligranulum and Eubacterium* all exceeded 1%, and we found seasonal effects on intestinal microbiota were more distinct in rural Mongolians^9^,^10^. From previous research, we found the intestinal microbiota of Mongolians exhibited some unique features. However, little is known about the functional gene and metabolic pathway of the intestinal microbiota of Mongolians^11^,^12^. It has been reported that prevalence of cardiovascular and cerebrovascular diseases in Mongolian adults was significantly higher than individuals of other ethnic groups and that these diseases became the leading cause of mortality in Mongolians. Some previous research attributes the aforementioned chronic metabolic diseases to the Mongolian dietary tradition of high consumption of red meat and liquor^1^. With the development of human microbiome project and next generation sequencing, great research effort has been dedicated to elucidating the relationship between intestinal microbiota and various human metabolic diseases^13^,^14^. The approach may also generate great insight by exploring the potential connection between cardiovascular and cerebrovascular diseases and the intestinal microbiota.

Here we characterized 110 healthy Mongolian adults gut microbiota by shotgun metagenomic sequencing and compared the intestinal microbiome among Mongolians, the Hans and European cohorts. (*i*) The taxonomic profile of intestinal microbiome among cohorts revealed the Actinobaceria and *Bifidobacterium* were the key microbes contributing to the differences among Mongolians, the Hans and Europeans at the phylum level and genus level, respectively. (*ii*) We constructed a non-redundant microbial catalogue for Mongolian contained 1,491,813 genes, and merged present catalogue with other published healthy gut microbial catalogues of the Hans and Europeans cohorts. The final merged non-redundant catalogue contained 5,127,164 genes, and 283,401 genes were common for all individuals. (*iii*) At the functional level, a unique microbial metabolic pathway profile was present in Mongolian’s gut which mainly distributed in amino acid metabolism, carbohydrate metabolism, energy metabolism, lipid metabolism, glycan biosynthesis and metabolism. We can attribute the specific signatures of Mongolian gut microbiome to their unique genotype, dietary habits and living environment.

## Results

### Taxonomic characterization

**Figure 2:**
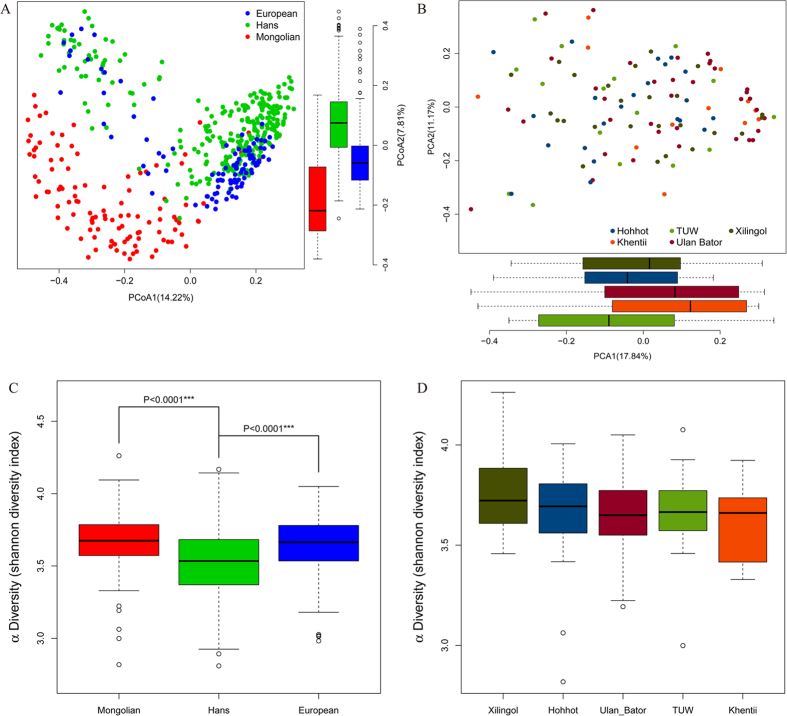
Comparison of the gut microbiomes of different area individuals. (**A**) PCoA plot with Bray–Curtis distances generated from species abundance of Mongolians, Europeans, and the Hans. (**B**) PCoA plot with Bray–Curtis distances generated from species abundance of the five arears Mongolian. (**C**) Shannon diversity index of Mongolians, Europeans, and the Hans at the species level. (**D**) Shannon diversity index of Mongolians, Europeans, and the Hans at the species level.

In present research, we characterized the fecal microbiota of 110 Mongolian individuals and compared the structure of intestinal microbiota among the cohorts from Hohhot, Xinlingol, TUW, Ulan Bator and Khentii. The sampling sites included a substantial part of relative densely populated places of ethnic Mongolian settlements, including areas in China and Mongolia. As a result, five representative Mongolian settlements were chosen to collect samples, including Ulan Bator city (35 healthy adults), TUW province (16 healthy adults) and the Khentii pasturing area (12 healthy adults) of Mongolia, Hohhot city (22 healthy adults) and Xilingol pasturing area (25 healthy adults) of China. Principal coordinates analysis (PCoA) based on the Bray–Curtis distance of species abundance (Fig. 2A) revealed the structure of intestinal microbiota between the Han Chinese’s and the European’s were similar, but significantly different from the Mongolian’s. The bacteria alpha diversity based on Shannon index (Fig. 2C) indicated the abundance of intestinal microbiota in Mongolian’s and European’s gut was significantly higher than Han Chinese’s. Importantly, we observed that there was no significant difference of these global parameters among the Mongolians located in Hohhot, Xinlingol, TUW, Ulan Bator and Khentii (Fig. 2D).

**Figure 3:**
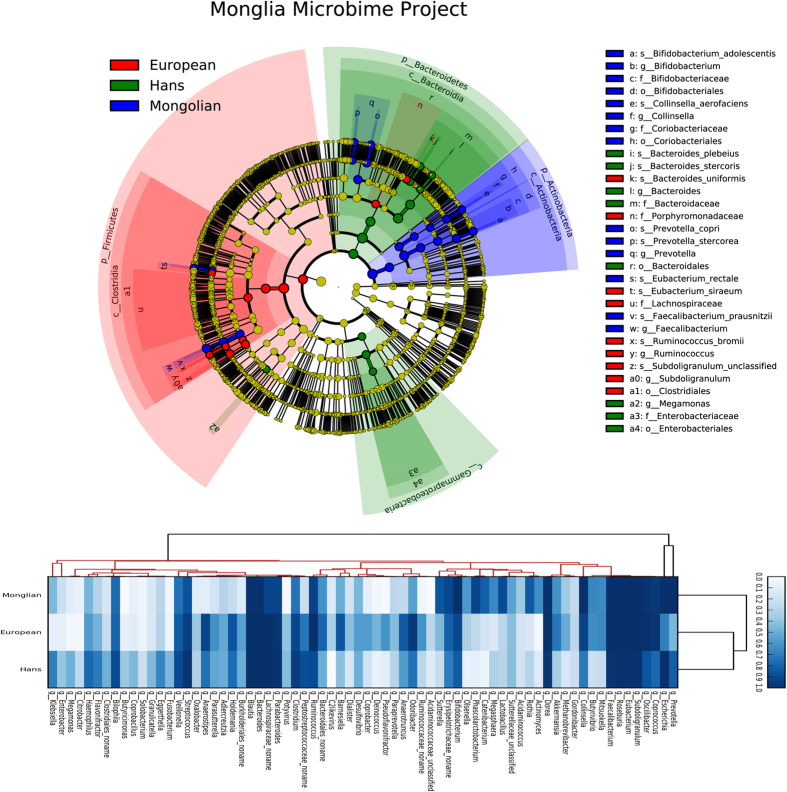
The Comparison of the Mongolian fecal microbiome project to others. All samples data about the three groups (European, Mongolia and Hans) have been detection taxonomic information by use Metaphlan2 programme, the cladogram derived from LEfSe analysis (http://huttenhower.org/galaxy/), the heatmap is the detection frequency for the microbial taxa in all samples (100% is deepblue, 0% is blank).

**Figure 4:**
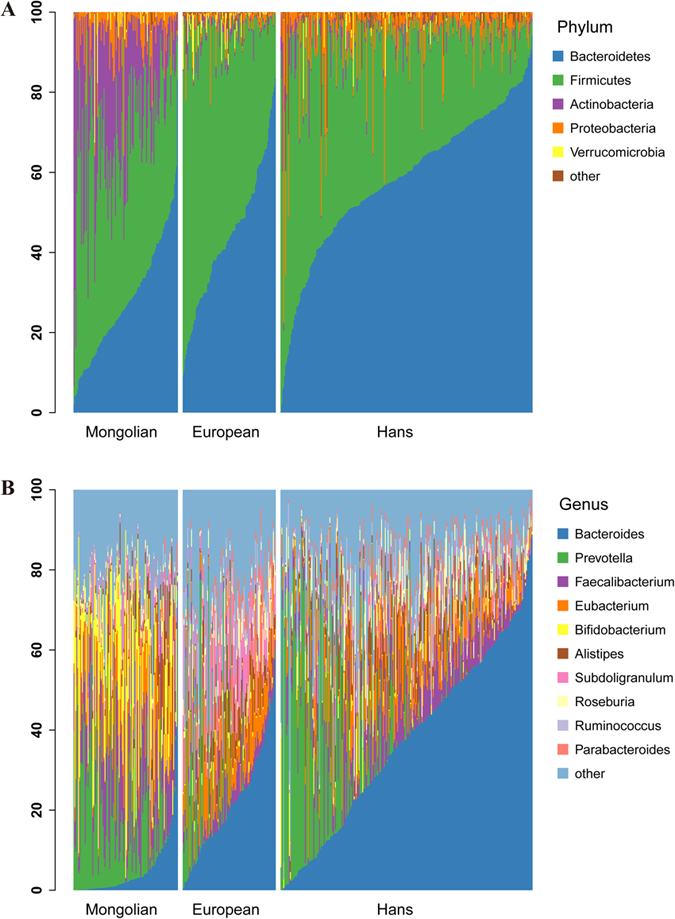
Relative taxa abundance comparison among three different area populations. (**A**) Phylum level. (**B**) Genus level. Individuals are showed along the horizontal axis, and relative taxa proportion is represented by the vertical axis.

To further explore the gut microbial community features of Mongolian population, we compared the results with that of previously reported samples^2^,^10^,^11^,^15^ and carried out taxonomic assignment for the metagenomic data using Metaphlan2 programme. Results were presented in the phylogenetic tree in Fig. 3. For all of these Mongolian subgroups, *Actinomyces, Rothia, Catenibacterium, Phascolarctobacterium, Sutterella and Lactobacillus* genera were remarkably enriched. On the contrary, *Holdemania, Peptostreptococcaceae, Potyvirus* or *Anaerostipes* were rarely detected in all Mongolian samples (Fig. 3). Taxonomic profile of the gut microbiomes among the populations (Fig. 4) also revealed that *Actinobaceria* and *Bifidobacterium* were the key components contributing to the differences among Mongolians, the Hans and Europeans at the phylum level and genus level, respectively.

### Metagenomic species (MGS) associated with Mongolians

To explore features of the gut microbiota in Mongolians, we compared the genes abundance in 110 Mongolians and 268 Hans, whereby we identified 115,786 genes with significant abundance differences (Wilcoxon rank-sum, Benjamin–Hochberg *q*-value < 0.001): 78,345 were more abundant in the Hans, and 37,438 in the Mongolians. Likewise, the similarity comparison between Mongolians and Europeans was also carried out, which identified 127,540 genes: 66,030 were enriched in the Europeans and 61,510 in the Mongolians.

**Figure 5:**
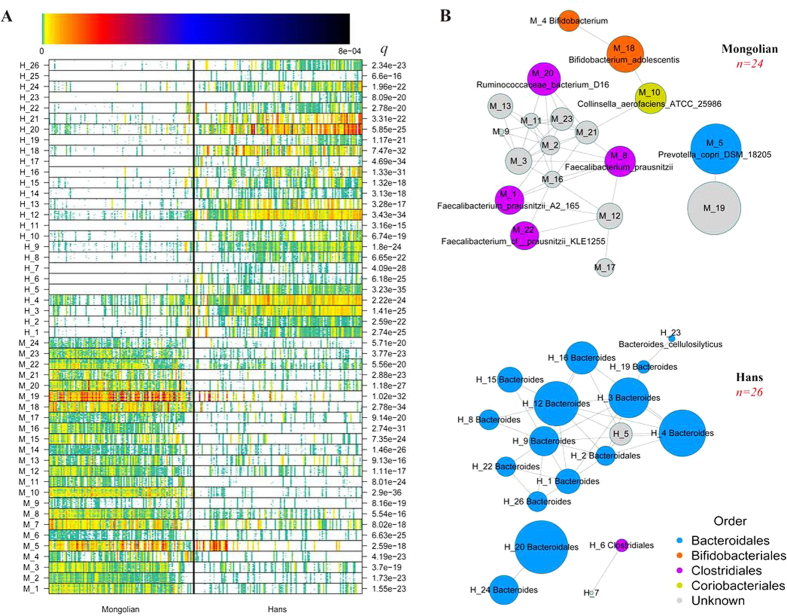
Gut MGS in the Mongolian and the Hans individuals. (**A**) The Heatmap of 25 ‘tracer’ genes abundance for each MGS in the Mongolians (110 individuals) and the Hans (268 individuals). Individuals are represented along the horizontal axis, sorted by increasing abundance of the Hans enriched MGS. Abundance of genes in rows is indicated by color gradient (white, not detected), and the enrichment significance is shown with Benjamin–Hochberg *q* value. (**B**) Co-occurrence network of enriched MGS in Mongolian (*n* = 24) and the Hans individuals (n = 26), respectively. Each node represents one MGS, and two nodes are linked if Spearman’s rank correlation > 0.6, using the edge width to represent the correlation strength. The node size is proportional to the mean relative abundance of MGS in the respective population. Nodes were colored based the phylogenetic order level.

The differentially abundant genes with high correlation were clustered into MGS^16^,^17^ based on their abundance in all samples. We found 50 MGS to be differentially abundant betweenthe two groups, 26 and 24 enriched in Hans individuals and Mongolian subjects, respectively (Fig. 5 and Supplementary Table 3). Based the taxonomic characterization of the MGS significantly enriched in Mongolians, we found three *Faecalibacterium prausnitzii*, two identified at strain level and one at specie level, which has anti-inflammatory properties and found to be associated with ‘healthy’ microbiome in previous reasearch^16^,^18^. One other MGS enriched in Mongolian was identified at species levels as *Coprococcus comes* ATCC 27758, which might contribute to gut health through butyrate production. Interestingly, *Collinsella aerofaciens,* which is enriched in Mongolians, was reportedly enriched in patients with symptomatic atherosclerosis^12^. For the comparison of Mongolians and Europeans, we found 29 and 16 MGS were enriched in European individuals and Mongolian subjects, respectively (Fig. S2). Remarkably, like the comparison between the Hans and Mongolians, *Faecalibacterium prausnitzii, Bifidobacterium adolescentis* and *Collinsella aerofaciens* were enriched in Mongolians.

**Figure 6:**
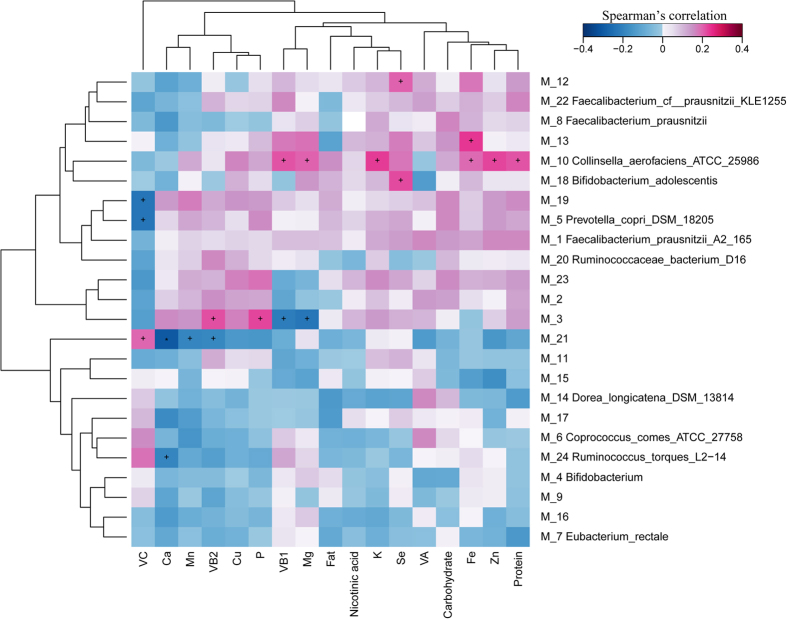
Numerical correlation between dietary indices and Mongolian enriched MGS in the comparison of Mongolians and the Hans. Spearman’s rank correlation coefficients is indicated by color gradient, red represents positive correlation; blue represents negative correlation. ^+^Denotes *P* < 0.05; *denotes *P* < 0.01.

Another intriguing question we focused on was the correlation between the Mongolians diet and their intestinal microbiota. By transforming food frequency questionnaire to the nutrition information, we calculated the Spearman’s rank correlation coefficient between the Mongolians-enriched MGS and the dietary nutrition factors (Fig. 6 and Fig. S3). From the figure, a significant positive (*P* < 0.05) correlation can be observed between the protein, K, Zn, Fe and VB1 and the species *Collinsella aerofaciens*. Additionally, the elements Se and Mg exhibited significant positive (*P* < 0.05) correlation with the species *Bifidobacterium adolescentis*.

### Gene catalogue of gut microbes

We sequenced the fecal metagenome of 110 Mongolian individuals using illumina platform and obtained 725 gigabases (Gb) raw data, with an average of 6.54 Gb for each sample. After quality control, we got an average of 6.33 Gb data for each, totalling 696 Gb of high-quality data which was free of adaptor and human DNA contaminants. Based on these data, we constructed a Mongolian gene catalogue distributed in five areas (Khentii pasturing area, TUW province, Ulan Bator, Hohhot City and Xilingol pasturing area) using the methodology developed by MetaHIT. Samples from Ulan Bator had the most abundant gene catalogue, containing 768,497 genes. Khentii set contained 400,178 genes, representing the fewest sample. Xilingol, Hohhot and TUW catalogue contained 512,067,702,521 and 433,515 genes, respectively. The merged non-redundant catalogue contained 1,491,813 genes. The Hohhot, Khentii, TUW, Ulan Bator and Xilingol catalogue sets contained 137,496, 99,760, 110,428, 277,758 and 242,637 unique genes, respectively.

Furthermore, we compared the Mongolian catalogue with four other published healthy gut microbial catalogues: ShenZhen (T2D), HangZhou (LC), American (HMP), European (MetaHIT)^2^,^10^,^11^,^15^. To facilitate this comparison, we predicted genes of the four healthy groups above using the same criteria. The Mongolian gene catalogue contained 1,418,578 genes, the American catalogue 2,243,320 genes, the European catalogue 1,711,862 genes, the HangZhou catalogue 1,183,368 genes and the ShenZhen catalogue 2,067,885 genes. In total, 283,401 genes were common to all catalogues. The Mongolian, American, European, HangZhou, and ShenZhen sets contained 599,370, 1015,117, 591,857, 233,036 and 777,465 unique genes, respectively. The final merged non-redundant catalogue contained 5,127,164 genes (Fig. S1).

### Functional characterization

**Figure 7:**
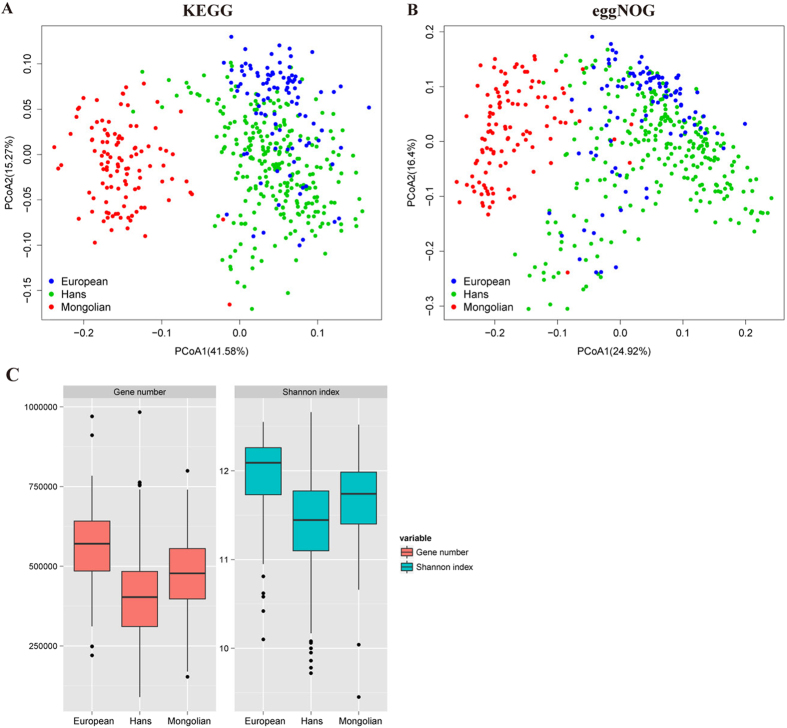
Function of Mongolian gut microbiomes distinctive to the European and the Hans population. (**A**) PCoA plot with Bray–Curtis distances generated from KEGG Orthologue (Enzyme) profiling. (**B**) PCoA plot with Bray–Curtis distances generated from eggNOG Orthologue profiling. (**C**) Gene diversity of Mongolians, Europeans, and the Hans.

**Figure 8:**
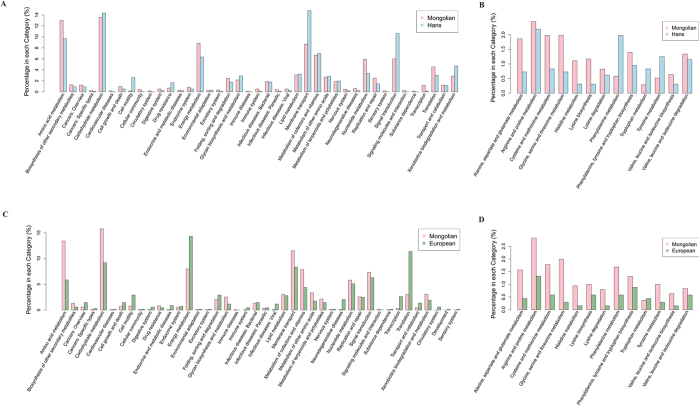
The distribution of KEGG functional categories of KO markers. (**A**) Comparison between the Mongolians-enriched and the Hans-enriched KO markers on level 2 of KEGG functional category. (**B**) Comparison between the Mongolians-enriched and the Hans-enriched KO markers on pathways in “Amino acid metabolism”. (**C**) Comparison between the Mongolians-enriched and Europeans-enriched KO markers on level 2 of KEGG functional category. (**D**) Comparison between the Mongolians-enriched and Europeans-enriched KO markers on pathways in “Amino acid metabolism”.

**Figure 9:**
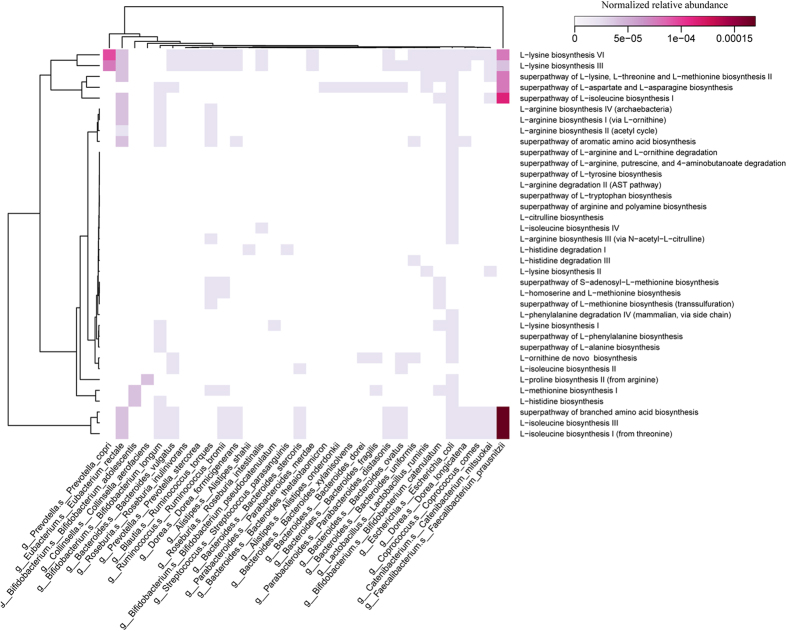
Heatmap of amino acid metabolic pathways of microbes in species level. The heatmap visualizes the abundance of each of the pathways in each of the species (median of Mongolians). The normalized relative abundance of each pathway of each specie was calculated by HUMAnN2 (http://huttenhower.sph.harvard.edu/humann2).

To explore the functional feature of the gut microbiota in Mongolian, we annotated the gene catalogue by KEGG and eggNOG. At the functional level, it can be observed the functional structures of intestinal microbiota among Mongolian, the Hans and Europeans were significantly different (Fig. 7A), as a unique KEGG orthologue group (KO) profile was present in Mongolian’s gut. Similarly to taxonomic diversity, Mongolian’s gut and European’s gut show higher diversity than Han Chinese’s in functional level (Fig. 7B). At pathway level, Mongolians enriched KOs was mainly distributed in amino acid metabolism, carbohydrate metabolism, energy metabolism, lipid metabolism, glycan biosynthesis and metabolism (Fig. 8A,C). In the amino acid metabolism, mainly enriched pathway were alanine, aspartate and glutamate metabolism, cysteine and methionine metabolism, lysine biosynthesis and lysine degradation (Fig. 8B,D). Combined the abundance analysis of amino acid metabolic pathways for each species, *Faecalibacterium prausnitzii, Prevotella copri* which were enriched in Mongolians, seemed to be the primary contributors to the amino acid metabolism, especially the L-lysine biosynthesis and L−isoleucine biosynthesis (Fig. 9). It potentially indicated some functional enrichment might be related to the enrichment of some specific species. The enriched amino acid metabolism and energy metabolism functions were associated with Mongolians’ high meat and fermentation uptake (Fig. S6). Moreover, we identified significantly different (reporter score > 1.6) abundant metabolic models between Mongolian and the Hans or Europeans by calculating the reporter scores. The majority of the models that were differentially abundant in the Mongolian subjects coincided with the observed changes in the metabolic pathway which mainly belong to the metabolic of carbohydrate, amino and nucleotide sugar, phosphotransferase and aminoacyl-tRNA and the biosynthesis of the methane and sulfide (Fig. S4).

Since we observed the carbohydrate metabolic between Mongolians and the Hans was significantly different. We further explored the potential for complex carbohydrate degradation in Mongolian and the Hans gut metagenomes, we screened for carbohydrate-active enzymes (CAZymes) in assembled contigs. As a result, Mongolian GM showed a higher diversity of CAZymes than that of the Hans GM (*P* < 0.0001, Wilcoxon rank-sum test; Fig. S5). This demonstrates an increased capacity for complex carbohydrate metabolism in the Mongolian GM.

## Discussion

In present research, we observed several distinct features present in the gut microbiota of ethnic Mongolian from two countries, Mongolia and China. Taxonomically, the genus *Collinsella* was significantly enriched in Mongolians, and a significant positive correlation was observed between protein uptaken in diet with this genus (Fig. 6). It was reported that the *Collinsella* was considered as the biomarker in symptomatic atherosclerosis patients^19^. In previous study, the intestinal microbiota of patients of symptomatic atherosclerotic and healthy controls were compared. In genus level, it is indicated the abundance of the *Collinsella* was increased in patients with symptomatic atherosclerosis, whereas *Roseburia* and *Eubacterium* were enriched in healthy controls^19^. Additionally, the *Collinsella, Enterobacteriaceae* and *Streptococcus* were considered as the potential pro-inflammatory GM components enriched in the T2D patients, which resulted in the rapid decline of the SCFA in gut^20^. Accordingly, we can infer the high protein uptake in Mongolians diet correlates with robust growth of the intestinal *Collinsella*, which may increase the risk of the morbidity of cardiovascular and cerebrovascular diseases in Mongolian adults.

On the other hand, the *Lactobacillus* and *Bifidobacterium* commonly considered as probiotics were largely detected in Mongolians gut^21^,^22^. As we known, the *Lactobacillus* and *Bifidobacterium* are major benefit microbes in human gut. They exhibit a range of health-promoting effects, including the regulation of intestinal microbial homeostasis^19^, the inhibition of pathogens in the intestinal mucosa^23^, the modulation of local and systemic immune responses, the production of vitamins^24^, and the bioconversion of a number of dietary compounds into bioactive molecules. Various species of *Lactobacillus* played a key role in yoghurt fermentation. And in previous study, researchers had demonstrated that the *Lactobacillus* was able to promote the colonization of *Bifidobacterium*^23^. Thus, the widely existence of the genera Lactobacillus and *Bifidobacterium* may be related to Mongolians dietary habits of fermented milk consumption. Additionally, we found that the genus *Bifidobacterium* exhibited significant positive correlation with the dietary selenium (Se). The interaction between the Se and the genus *Bifidobacterium* had been reported in previous study, and the supplementary Se in MRS ager was able to promote the growth of the *Bifidobacterium*^25^.

The increased pathway abundance of carbohydrate, amino acid and nucleotide sugar, and phosphotransferase and aminoacyl-tRNA of intestinal microbiota in Mongolians suggested the more vigorous microbial growth and metabolic stage in Mongolian’s gut compared with that of Han Chinese. The aminoacyl-tRNA was able to combine with its corresponding animal acid, and transferred the animal acid to the ribosome^26^. In present research, we found the biosynthesis of aminoacyl-tRNA was enhanced which indicated an overall enhanced level of microbial gene expression in the gut of Mongolians. Moreover, the up-regulation of the metabolic pathway of phosphotransferase provide the source of energy for microbe’s proliferation^27^. On one hand, the robust metabolism of gut microbes in Mongolians was able to promote the digestion and absorption of food in intestine. On the other hand, attributed to the stronger metabolism, intestinal microbes would produce more metabolic products including beneficial or harmful factors which had a greater impact on human health.

Accordingly, we found that the biosynthesis of the methane and sulfide in intestinal microbiota of Mongolians were significantly higher than that of other populations. The two compounds were recognized as the typical inflammatory factor in human gut. Previous researches had demonstrated the perniciousness of the methane and sulfide in intestine of T2D^28^ and IBD patients^29^,^30^. As such, it is tempting to speculate that the high morbidity of cardiovascular and cerebrovascular diseases in Mongolian adults were pertinent to their relatively strong biosynthesis of the methane and sulfide in intestinal microbiota.

In Mongolian diet, meat (including mutton and beef) is the major source of protein, and the Mongolians consume a significantly greater amount of animal protein than Han Chinese. Intestinal microbiota played the key role in animal protein metabolism. Within this microflora, the genera *Clostridium* and *Bacteroides*, which were extensively detected in the Mongolians gut, have been mainly associated with the metabolism of animal proteins, a variety of amino acids and saturated fat acids^16^,^17^,^18^. Accordingly, we noticed the pathways for degradation of protein and the biosynthesis of the amino acid were enriched in the intestinal microbiota of Mongolians.

## Materials and Methods

### Sample information

In present research, a total of 110 Mongolians were recruited for sampling. Among these participants, 63 Mongolians were from Mongolia and the other 47 were from Inner Mongolia of China. For participants from Mongolia, 35 volunteers lived a typical modern lifestyle in Ulan Bator, the capital of Mongolia, 16 volunteers lived in the TUW province which covers the suburbs of Ulan Bator, and 12 volunteers lived in the Khentii pasturing area, a typical Mongolian steppe. For the 47 volunteers from Inner Mongolian of China, 22 volunteers lived in Hohhot, the capital of Inner Mongolia, which lived an urban lifestyle. The others lived in Xilingol pasturing area, a typical Mongolian grassland like Khentii. After obtaining the written informed consent, we collected habitual long-term dietary information from all participants using a food frequency questionnaire and transformed them to the nutrition information (Supplementary Table 1). The study protocol was approved by the Ethical Committee of the Inner Mongolia Agriculture University (Hohhot, China). Sampling and all subsequent steps described in the Materials and Methods have been conducted in accordance with the approved guidelines.

### DNA sequencing

DNA was extracted from faecal samples using the QIAampDNA Stool MiniKit as previously described^10^. The metagenomic DNA libraries were constructed with 2 μg genome DNA according to the Illumina TruSeq DNA Sample Prep v2 Guide, with an average of 500 bp insert size. The quality of all libraries was evaluated using an Agilent bioanalyser with a DNA LabChip 1000 kit. Sequencing was performed by Illumina Hiseq2500.

### Metagenomic data for comparison

The public gut microbial metagenomic data used in this study include: (i) 83 healthy Hans fecal samples (Hangzhou), which were downloaded from EBI under accession number ERP005860^14^; (ii) 185 healthy Hans fecal samples (Shenzhen), which were downloaded from NCBI under accession number SRA045646^14^; (ii) 99 healthy European fecal samples from the MetaHIT project, which were downloaded from EBI under accession number ERA000116^2^.

### Quality control of reads

Raw paired-end reads were processed quality control using the following criteria: (1) reads with adaptor were removed; (2) reads were trimmed from the 3′ end using a quality threshold of 30; (3) reads containing more than 50% bases with low quality (Q30) were removed; (4) Reads short than 70 bp were removed; (5) reads that mapped to human genome were removed. For alignment, SOAPaligner 2.21 27 was used by parameter of ‘-m 100 -x 1000’, which are “reporting all repeat hits, Minimal insert size of 100 bp, Maximal insert size of 1000”. For detailed information on the meaning of each option above, we refer to http://soap.genomics.org.cn/soapaligner.html. The resulting high-quality reads were used for the further analysis.

### De *novo* assembly and gene catalogue construction

High-quality reads were used to assembly with SOAPdenovo 28 (version 2.04) using parameters of ‘-M 3 -u -L 100 -d 1 –F –k 53’, which are “mergeLevel of 3, un-masking contigs with high/low coverage before scaffolding, minContigLen of 100 and KmerFreqCutoff of 1, filling gaps in scaffold, k-mer length of 53”. For detailed information on the meaning of each option above, we refer to http://soap.genomics.org.cn/soapdenovo.html. The resulting scaffolds were cut into contigs at ambiguous Ns and the contigs longer than 500 bp were saved. All these contigs were applied for gene prediction.

We use MetaGeneMark^31^ (version 3.26) to identify ORFs from the contigs of each sample using a length threshold of 100 bp (Supplementary Table 2). Then the non-redundant gene catalogue was constructed by pairwise comparison of all the predicted ORFs with CD-HIT^32^ (version 4.5.7) and the redundant genes were removed using a sequence identity cut-off of 0.95 and aligned length covered over 90% of the shorter sequence. The final non-redundant Mongolian gene catalogue contains 1,491,813 microbial genes, with an average length of 808 bp.

In light of some low-abundance microbes that were not detected in the limited sequencing data, we combined with the previously constructed gene catalogue, including MetaHIT gene catalogue^2^, HMP gene catalogue^3^, T2D gene catalogue^14^ and LC gene catalogue^13^ to build a non-redundant gene catalogue for further analyses. The ORFs in the contigs of healthy individuals from above studies were predicted using MetaGeneMark and merged with Mongolian gene catalogue into a final one with the same criteria of 90% coverage of shorter gene and 95% identity. Finally, we got a non-redundant human gut gene catalogue which contains 4,998,380 genes, with an average length of 763 bp.

### Taxonomic and gene profiling

We use MetaPhlAn2^33^ to produce organism abundance profiling with default parameters, which relied on about 1 million unique clade-specific marker genes identified from about 17,000 reference genomes. 1,036 microorganisms were mapped to the marker genes, include 9 Archaea, 963 Bacteria, 18 Eukaryota and 46 Viruses.

Relative abundances of the genes were determined with the procedure introduced in Qin N *et al*. Nature 2014^13^. When calculating the abundance of genes, the high quality reads from each sample were aligned against the gene catalogue by using SOAPalign 2.21^15^ with parameters of ‘-r 2 -m 100 -x 1000’ and only the both paired-end reads which could be mapped to a same gene were accepted. For alignment, SOAPaligner 2.21 was used by parameter of ‘-m 100 -x 1000’, which are “reporting all repeat hits, Minimal insert size of 100 bp, Maximal insert size of 1000”. For detailed information on the meaning of each option above, we refer to http://soap.genomics.org.cn/soapaligner.html.

### MGS analysis

For the comparison of the faecal microbiome between Mongolians and the Hans, gene markers with differentially abundance were identified (Benjamin–Hochberg *q*-value < 0.001). The comparison between Mongolians and Europeans was carried out in the same way. To cluster genes into Metagenomic Species (MGS), we followed the method described by Le Chatelier^33^ and Nielsen^15^. We clustered the genes with Spearman correlation coefficient (rho) > 0.8 using single-linkage clustering and then fused the clusters with more than 25 genes which had a Spearman correlation coefficient (rho) >0.8.

The taxonomically annotation of MGS were performed as previously describe^13^. MGS was assigned to a taxonomical level from strain to super kingdom level when >90% of its genes had a best hit to the same phylogenetic group using blast with >95% identity and >90% overlap of query.

To constructed the co-occurrence network of MGS, we computed the Spearman correlation coefficient between MGS using their abundances and clustered the MGS according to the Spearman’s correlation. The co-occurrence network of MGS was then visualized by Cytoscape3.0.2.

### KEGG, EggNOG and CAZy analysis

Putative amino acid sequences of the predicted genes were aligned against the proteins in eggNOG 3.0 database^34^ and KEGG database^35^ (release 2014-12-09) using BLAST. Each gene was assigned to one or more OG(s) or EC(s) by the highest annotated scoring hit(s) containing at least one HSP scoring over 60 bits. To construct the profiling of OGs in eggNOG and ECs in KEGG database, we accumulated the relative abundance of genes from the same OG or EC using the methods introduced in Qin J. *et al*. Nature 2010^2^. To identify the differentially enriched KO modules, we computed their reporter scores from the Z-scores of individual KOs^36^. A module with a reporter score of Z > 1.6 was defined as differentially enriched module.

CAZymes (Carbohydrate-Active Enzymes) were predicted from amino acid sequences by against to family-specific HMM of CAZymes in dbCAN database^37^ using Hmmscan program in HMMER 3.0 package^38^.

The raw sequencing data for all samples have been deposited to Sequence Read Archive (http://www.ncbi.nlm.nih.gov/sra/) under accession SRP080787.

## Additional Information

**How to cite this article**: Liu, W. *et al*. Unique Features of Ethnic Mongolian Gut Microbiome revealed by metagenomic analysis. *Sci. Rep.* **6**, 34826; doi: 10.1038/srep34826 (2016).

## Supplementary Material

Supplementary Information

Supplementary Information

## Figures and Tables

**Figure 1:**
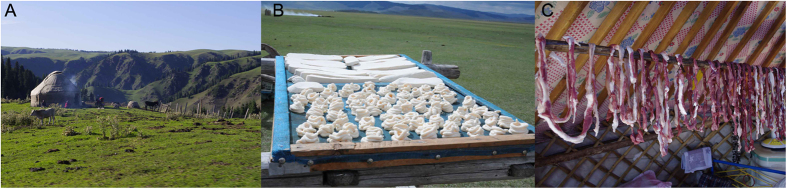
Country-wide sampling highlights distinct features of Mongolia gut microbiota. (**A**) Traditional Mongolian yurt in pasturing area. (**B**,**C**) Mongolian’s staple foods of such as cheese and red meat are drying in the air.
