# Corrigendum: Unique Features of Ethnic Mongolian Gut Microbiome revealed by metagenomic analysis

**DOI:** 10.1038/srep39576

**Published:** 2017-01-04

**Authors:** Wenjun Liu, Jiachao Zhang, Chunyan Wu, Shunfeng Cai, Weiqiang Huang, Jing Chen, Xiaoxia Xi, Zebin Liang, Qiangchuan Hou, Bin Zhou, Nan Qin, Heping Zhang

Scientific Reports 6: Article number: 3482610.1038/srep34826; published online: 10 06 2016; updated: 01 04 2017

The original version of this Article contained errors in the spelling of the authors Bin Zhou, which was incorrectly given as Bing Zhou, and Xiaoxia Xi, which was incorrectly given as Xiaoxia XI.

This Article also contained typographical errors in the Results section under the subheading ‘Metagenomic species (MGS) associated with Mongolians’.

“whereby we identified 115,786 genes with significant abundance differences”.

now reads

“whereby we identified 115,783 genes with significant abundance differences”.

In addition, under the subheading “Functional characterization”.

“At the functional level, it can be observed the functional structures of intestinal microbiota among Mongolian, the Hans and Europeans were significantly different (Fig. 7A), as a unique KEGG orthologue group (KO) profile was present in Mongolian’s gut. Similarly to taxonomic diversity, Mongolian’s gut and European’s gut show higher diversity than Han Chinese’s in functional level (Fig. 7B)”.

Now reads

“At the functional level, it can be observed the functional structures of intestinal microbiota among Mongolian, the Hans and Europeans were significantly different (Fig. 7B), as a unique KEGG orthologue group (KO) profile was present in Mongolian’s gut. Similarly to taxonomic diversity, Mongolian’s gut and European’s gut show higher diversity than Han Chinese’s in functional level (Fig. 7C)”.

Finally, Figure legend 2D.

“Shannon diversity index of Mongolians, Europeans, and the Hans at the species level”.

now reads:

“Shannon diversity index of the Mongolians located in Hohhot, Xinlingol, TUW, Ulan Bator and Khentii at the species level”.

These errors have now been corrected in the PDF and HTML versions of the Article.

