# Supplementary material for: Unique Features of Ethnic Mongolian Gut Microbiome revealed by metagenomic analysis
Source: Sci Rep. 2016 Oct 6;6:34826. doi: 10.1038/srep34826 (PMC5052615; doi:10.1038/srep34826)
Supplement: Supplementary Information [file srep34826-s1.pdf]

Supplementary Figures

Unique Features of Ethnic Mongolian Gut Microbiome revealed by metagenomic analysis

Wenjun Liu[1*], Jiachao Zhang[1*], Chunyan Wu[2*], Shunfeng Cai[2], Weiqiang Huang[1], Jing Chen[2], Xiaoxia XI[1], Zebin Liang[2], Qiangchuan Hou[1], Bing Zhou[2], Nan Qin[3]†, Heping Zhang[1]†

The supplementary imformation includes:

Supplementary Figures S1-S6

Supplementary Figures

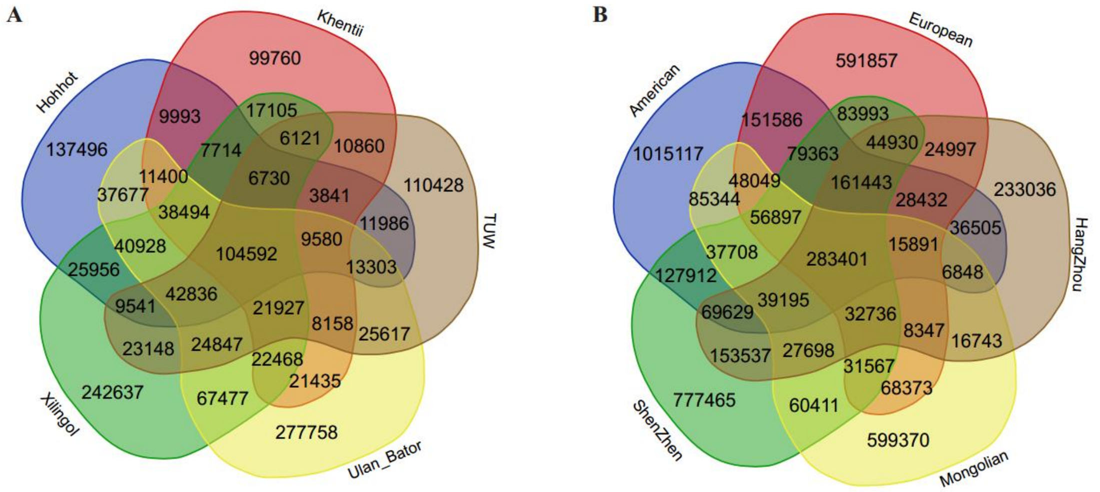

Figure S1 | Comparison of the current major human microbiome gene sets by venn diagram. (A) Microbiome gene sets venn diagram of the Mongolians distributed in five areas (Khentii pasturing area, TUW province, Ulan Bator, Hohhot City and Xilingol pasturing area). (B) Microbiome gene sets venn diagram of the Mongolians, healthy individuals from ShenZhen, healthy individuals from HangZhou, the Americans (healthy individuals from HMP) and the Europeans (healthy individuals from MetaHIT). The total gene number in each gene set and the overlapping areas are indicated.

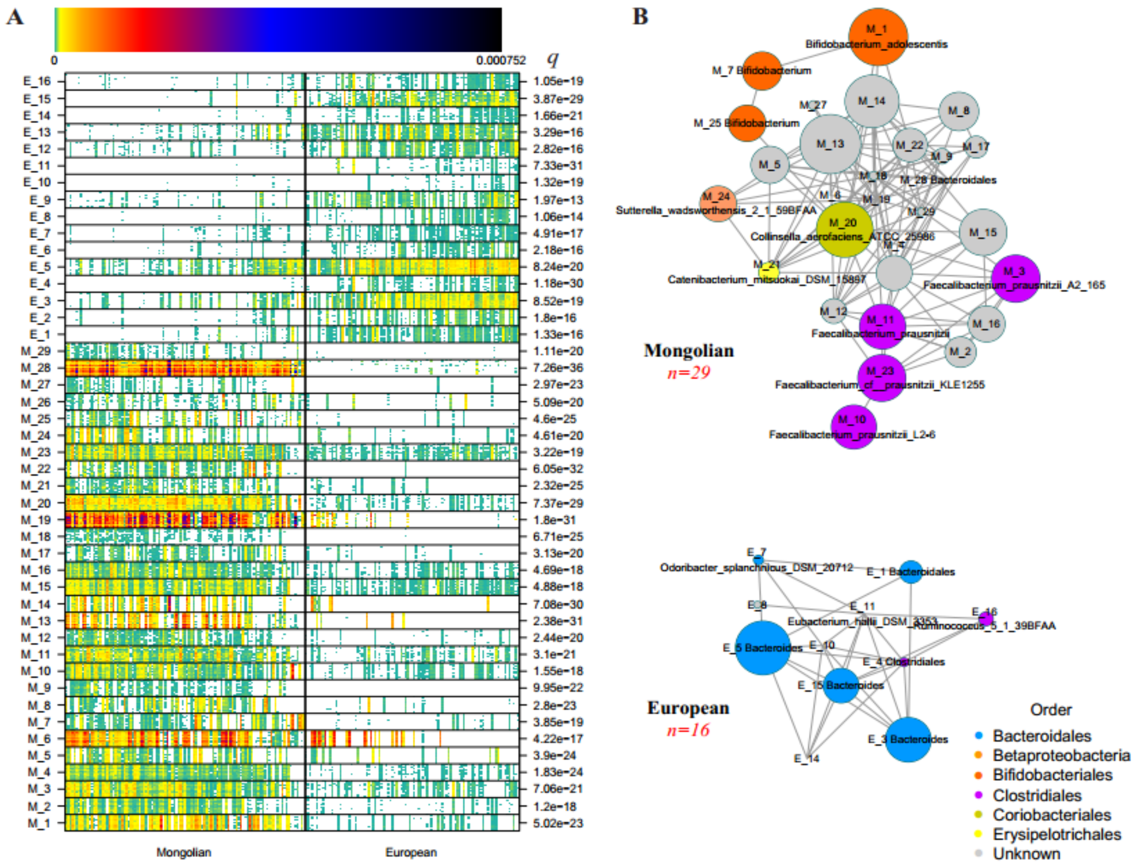

Figure S2 | Gut MGS in the Mongolian and European individuals. (A) The Heatmap of 25 'tracer' genes abundance for each MGS in the Mongolians (110 individuals) and the Europeans (99 individuals). Individuals are represented along the horizontal axis, sorted by increasing abundance of the European enriched MGS. Abundance of genes in rows is indicated by color gradient (white, not detected), and the enrichment significance is shown with Benjamin–Hochberg *q* value. (B) Co-occurrence network of enriched MGS in the Mongolian (*n*=24) and European individuals (n=26), respectively. Each node represents one MGS, and two nodes are linked if Spearman's rank correlation >0.6, using the edge width to represent the correlation strength. The node size is proportional to the mean relative abundance of MGS in the respective population. Nodes were colored based the phylogenetic order level.

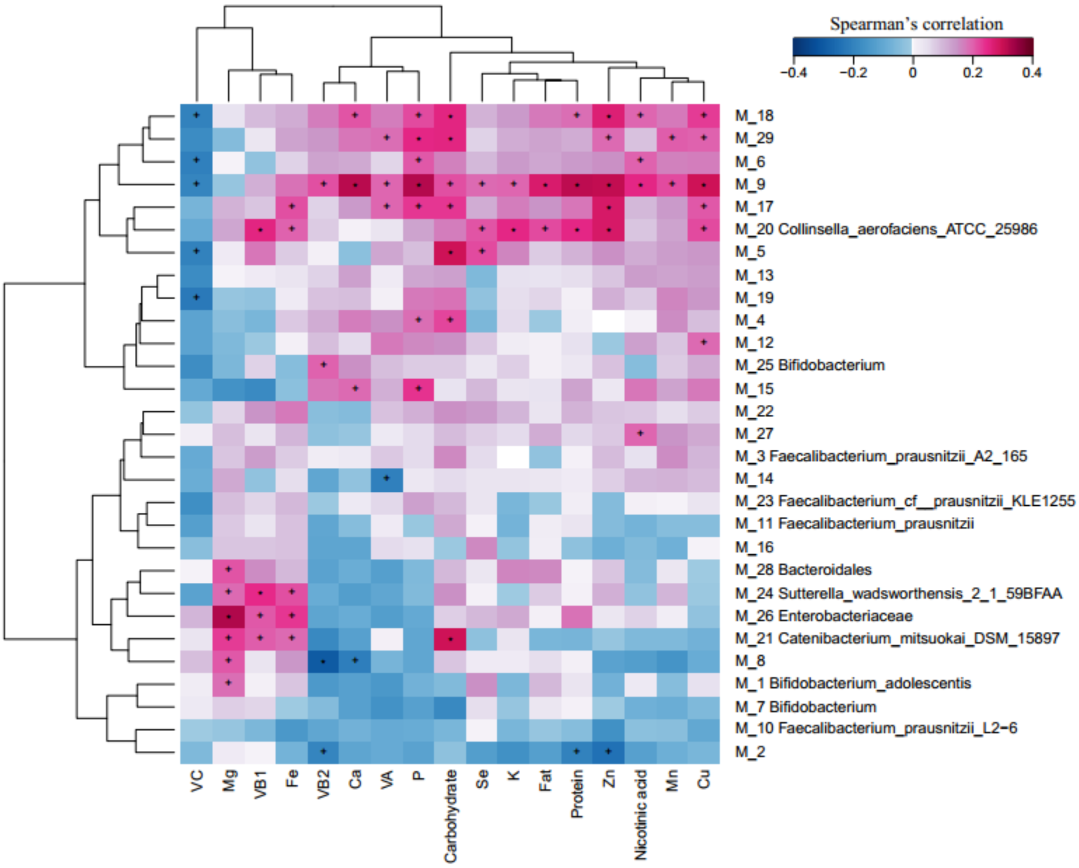

Figure S3 | Numerical correlation between dietary indices and Mongolian enriched MGS in the comparison of Mongolians and Europeans. Spearman's rank correlation coefficients is indicated by color gradient, red represents positive correlation; blue represents negative correlation. '+' denotes *P*<0.05; '*' denotes *P*<0.01.

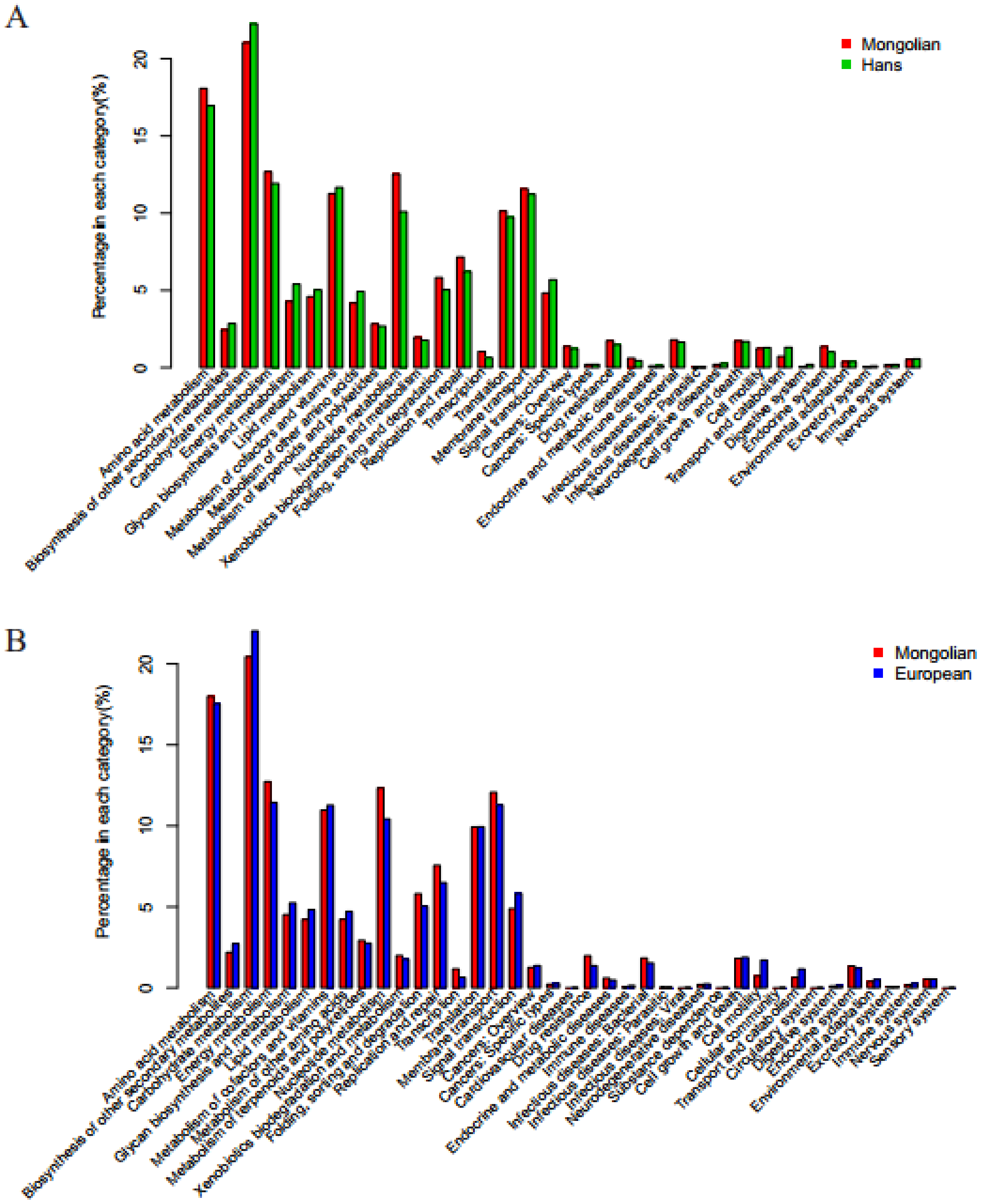

Figure S4 | KO modules overrepresented in Mongolian, European or the Hans individuals. The relative abundances of KO modules were compared between Mongolians and Europeans or the Hans, and modules with a significant difference in reporter score both in the two compares (<-1.6, enriched in former; >1.6, enriched in latter) are shown. +, reporter score <-2.3 or >2.3.

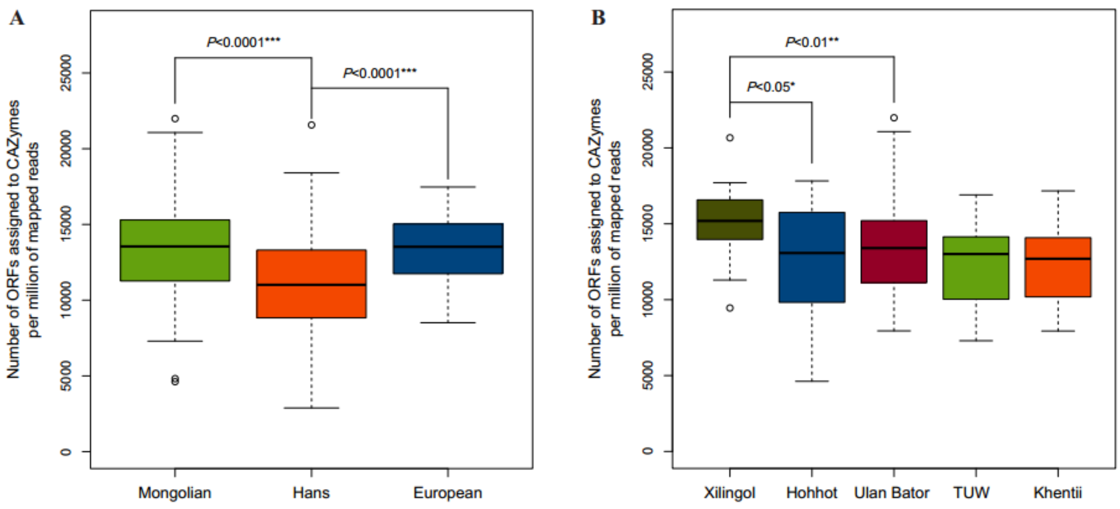

Figure S5 | Comparison of CAZymes diversity. The Gut Microbiomes Open reading frames (ORFs) were screened for CAZymes. For each sample, the total number of ORFs assigned to CAZymes were normalized by read count. Significance was calculated by Wilcoxon rank-sum test. (A) The Mongolian, the European, and the Hans. (B) The Mongolians distributed in five areas (Khentii pasturing area, TUW province, Ulan Bator, Hohhot City and Xilingol pasturing area).

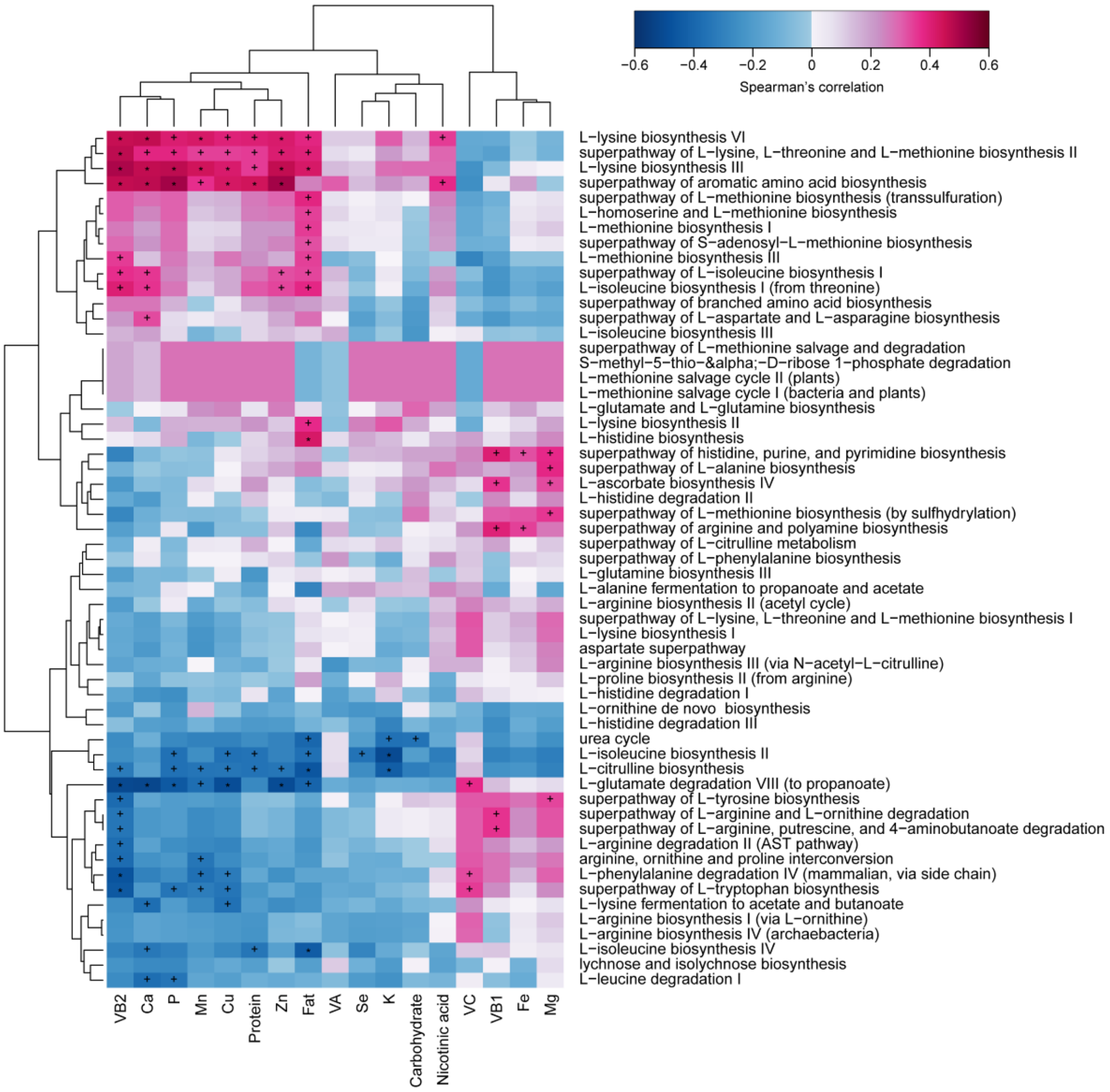

Figure S6 | The enriched amino acid metabolism and energy metabolism functions were associated with Mongolians' high meat and fermentation uptake.
